# Report of two rare manifestations of abdominal tuberculosis mimicking neoplasia

**DOI:** 10.1093/jscr/rjac079

**Published:** 2022-03-30

**Authors:** Senta Faulhaber, Sebastian Schindera, Mark Hartel, Alexander Gräfitsch

**Affiliations:** Department of Visceral Surgery, Kantonsspital Aarau, Aarau, Switzerland; Department of Radiology, Kantonsspital Aarau, Aarau, Switzerland; Department of Visceral Surgery, Kantonsspital Aarau, Aarau, Switzerland; Department of Visceral Surgery, Kantonsspital Aarau, Aarau, Switzerland

**Keywords:** abdominal tuberculosis, tuberculous peritonitis, pancreatic tuberculosis, pseudotumor, intestinal, obstruction, surgery

## Abstract

Abdominal tuberculosis (TB) can affect any organ of the gastrointestinal tract, and as a result of its unspecific symptoms, it may even mimic neoplasia. Rare manifestations are difficult to detect even for the trained eye and require clinical suspicion. We report rare cases of a mechanical ileus due to peritoneal TB in a 41-year-old man and an isolated peripancreatic infection in a 54-year-old woman. While in one patient, suspected malignancy led to diagnostic laparoscopy, it led to a total pancreatectomy with splenectomy in the other case. However, both times histology ruled out malignancy and showed unexpected similarities with TB. The patients responded well to medical treatment, although one patient is struggling with pancreatogenic diabetes.

## INTRODUCTION

Abdominal tuberculosis (TB) accounts for 10% of extrapulmonary manifestations and can affect all organs of the gastrointestinal tract [1, 2]. Symptoms are non-specific, vary depending on the location and can mimic those of malignant or chronic inflammatory diseases [1, 3]. Thus, correct diagnosis is challenging, leading to delayed recognition and increased morbidity and mortality [1–3].

We report on two cases presenting with rare manifestations of abdominal TB at our unit.

## CASE 1

**Figure 1:**
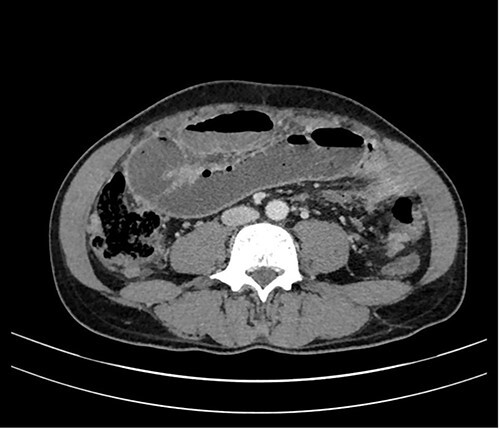
CT axial view: peritoneal thickening and mechanical ileus.

In a previous healthy 41-year-old male with a 3-month history of abdominal pain, fever, fatigue, night sweats and weight loss of 5 kg, a computed tomography (CT) of the abdomen revealed a mechanical ileus due to suspected distinct peritoneal carcinomatosis in the entire abdomen of an unknown primary (Fig. 1).

The patient, originally from Eritrea, had lived in Switzerland for 5 years. The patient had no previous surgeries, and a quantiferon test (QFT) in 2017 was positive.

On palpation, there was a tenderness in the entire abdomen. Notable laboratory findings were: hemoglobin of 122 g/l (135–172 g/l), serum amylase of 61 U/l (13–53 U/l) and c-reactive protein of 62.2 mg/l (<3.0 mg/l). After conservative therapy with a nasogastric tube, intravenous fluids and laxatives failed, we went for diagnostic laparoscopy.

**Figure 2:**
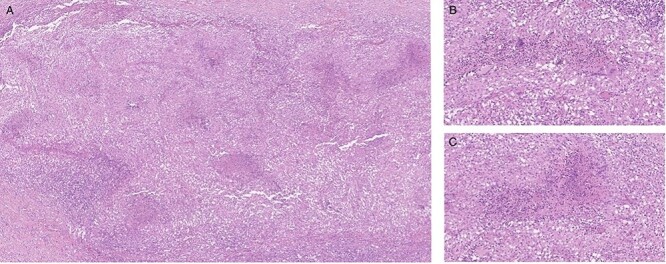
Histological finding of peritoneal biopsies showing extended granulomatous inflammation; necrosis in the center id multiple epithelioid granulomas; several multinucleated giant cells; hematoxylin and eosin staining, 5× magnification (**A**), 20x magnification (**B**, **C**).

Intraoperatively, in addition to numerous metastasis-suspected nodules on the peritoneum, we found dense adhesions of the omentum to the small intestine. After biopsies and cytology were gained, we finished the procedure without further interventions. Histopathology revealed granulomatous peritonitis with necrosis and multinuclear giant cells consistent with TB (Fig. 2).

However, polymerase chain reaction (PCR) could not detect mycobacterial deoxyribonucleic acid, while material for culture detection has not been collected intraoperatively. A subsequent test for human immunodeficiency virus (HIV) was negative. Due to the suggestive findings for TB, and the previously positive QFT, we initiated an intravenous antibiotic therapy with amikacin, moxifloxacin and rifampicin. Once the ileus had resolved, we switch to oral therapy (rifampicin, isoniazid, pyrazinamide and ethambutol), and the patient was discharged on Day 14. The antibiotic course was finished after 6 months at which point the patient was completely asymptomatic, tolerated normal oral diet and had regular bowel movements.

## CASE 2

**Figure 3:**
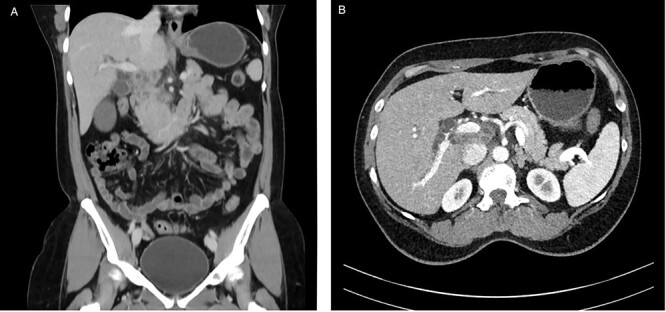
CT with contrast (**A**) coronal view and (**B**) axial view: cystic solid mass in the region of the hepatic hilus with unclear association to the head of the pancreas.

A 53-year-old female of Indian origin, resident in Switzerland for 20 years, presented with diffuse abdominal pain, nausea and meteorism in the primary care consultation. After an initial workup with blood tests and ultrasound (US), a CT of the abdomen was performed (Fig. 3), which revealed enlarged, central necrotic lymph nodes adjacent to the common bile duct (CBD) and the pancreatic head. The enlarged lymph nodes caused an obstruction of the CBD. The patient was referred to our institution for further diagnostic tests.

**Figure 4:**
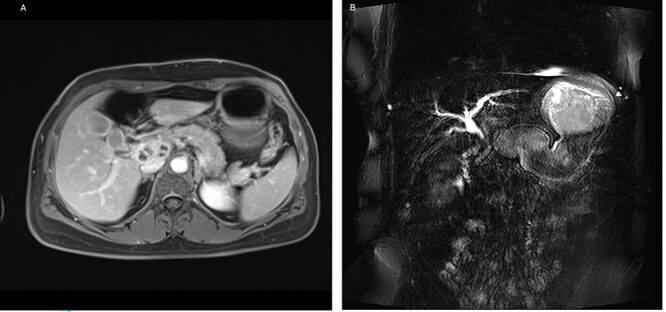
(**A**) Axial-T1w view with contrast: findings correlating with the CT and (**B**) magnetic resonance cholangiopancreatography showing a signal interruption of the CBD due to obstruction.

On admission, the patient seemed to be in good health, and her previous medical history contained no surgery. She reported pruritus, loss of appetite and weight loss of 5 kg in the past 3 months. Clinical examination showed slight epigastric tenderness and scleral icterus. Except for elevated liver function tests, the other blood tests, including tumor markers AFP, CEA and CA 19-9, were normal. For further assessment, we performed a magnetic resonance imaging of the abdomen, which confirmed the findings of the CT (Fig. 4).

After discussion in our multidisciplinary team meeting, we assumed that the pancreatic mass would most likely be a malignant tumor, as it obstructed the CBD. Consequently, we decided to do an explorative laparotomy after preoperative stent placement by an endoscopic retrograde cholangiopancreatography.

**Figure 5:**
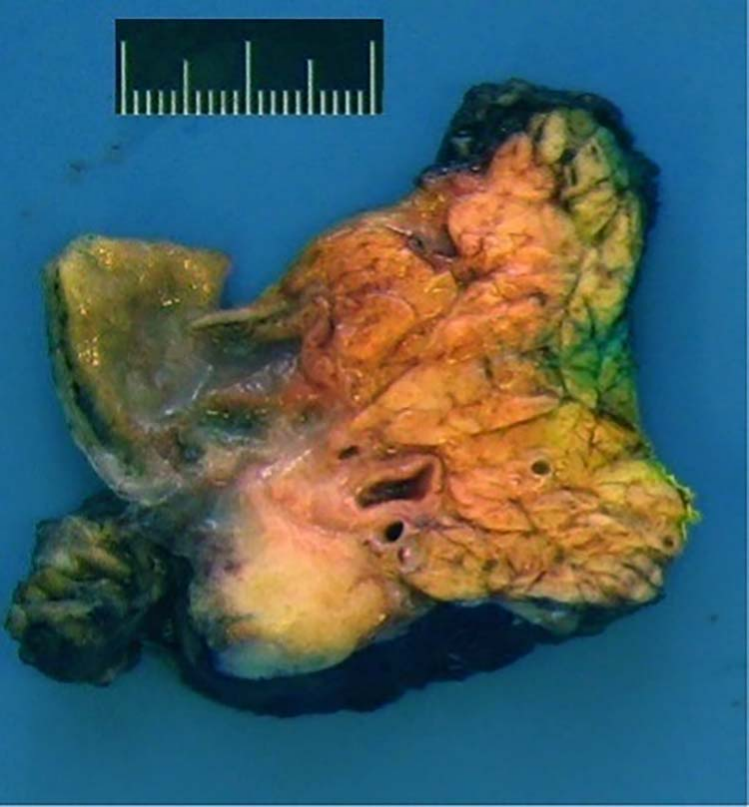
Macroscopic view: anterior surface of the pancreatic head (6 x 4 x 3.5 cm size).

Intraoperatively, we found a tumor infiltrating the duodenum and the portal vein as well as lymphadenopathy in the hepatoduodenal ligament. Therefore, we performed a total pancreatectomy with a splenectomy in an oncologic intention. We did not attempt a partial duodenopancreatectomy as the pancreatic tissue seemed too soft for a safe anastomosis. Our pathologists found a granulomatous inflammation around the pancreatic duct and in one lymph node (Fig. 5).

PCR confirmed an infection with *Mycobacterium tuberculosis* complex, while a chest X-ray and HIV serology were normal. We treated the patient with a combination of rifampicin, isoniazid, ethambutol and pyrazinamide. The patient tolerated the antimicrobial therapy well; however, a lymphatic fistula prolonged her post-operative course and she developed pancreatogenic diabetes.

## DISCUSSION

In developed countries, abdominal TB is a rare disease, although its incidence is increasing. Most patients have an immigrational background or are immunocompromised, especially because of HIV [1, 3, 4].

Most frequently, it affects the peritoneum (66%), the mesenteric lymph nodes (62%) and the small intestine (33%), particularly the ileocecal region [2–4]. On the contrary, isolated pancreatic TB occurs rarely (5%). As in our patient, it may hardly be distinguished from a malignant disease [2, 5–7]. In general, abdominal TB is caused by the reactivation of pulmonary infection with *M. tuberculosis*. The bacteria can spread not only via lymphatic or blood vessels but also directly from infected organs [2–4, 8].

Peritoneal TB mostly causes gradually increasing mild symptoms, and pancreatic TB may even be asymptomatic unless abscesses or masses occur [4–6, 8]. The most frequent symptoms of a peritoneal infection are ascites and abdominal tension [3, 4, 8]. Mechanical ileus due to adherent small bowel loops or inflammation of the omentum majus is rare [4, 9, 10]. In pancreatic TB, epigastric pain and jaundice as a result of extrahepatic cholestasis are typical [5–7].

Regarding peritoneal TB, US may detect ascites or omental thickening and CT scans may reveal thickened small intestinal loops [2, 8, 10]; finally, laparoscopy can confirm the diagnosis [1, 4, 10]. In case of pancreatic involvement, specimens can be obtained both by CT and US-guided fine needle biopsy [5, 6]. In most cases, the diagnosis is made by a combination of several diagnostic tools, including the detection of serum ribonucleic acid or histopathological evaluation [1–3]. Gaining samples for both microbiology and histopathology is crucial [2, 4]. As we did not send, due to suspected malignancy, material for microbiological testing, it was impossible to definitively confirm TB in the first presented case.

The treatment of abdominal TB contains a 6-month course of antibiotics and should be started if clinical suspicion exists, even if cultural results are pending [1, 2]. Limited surgical procedures are reserved for the treatment of complications, which occur in ~15% [1, 3, 8].

## CONFLICT OF INTEREST STATEMENT

None declared.

## FUNDING

None.
